# Association between monocyte to lymphocyte ratio and diabetic foot ulcer in the population of the US with diabetes based on the 1999-2004 National Health and Nutrition Examination Survey data: a retrospective cross-sectional study

**DOI:** 10.3389/fendo.2024.1361393

**Published:** 2024-04-24

**Authors:** Zirui Li, Yang Jian, Zairong Wei

**Affiliations:** ^1^Department of Burns and Plastic Surgery, Affiliated Hospital of Zunyi Medical University, Zunyi, Guizhou, China; ^2^The Collaborative Innovation Center of Tissue Damage Repair and Regeneration Medicine of Zunyi Medical University, Zunyi, Guizhou, China

**Keywords:** diabetic foot ulcer, monocyte-lymphocyte ratio, diabetes, NHANES, inflammation

## Abstract

**Background:**

Diabetic foot ulcer (DFU) is a severe complication that occurs in patients with diabetes and is a primary factor that necessitates amputation. Therefore, the occurrence and progression of DFU must be predicted at an early stage to improve patient prognosis and outcomes. In this regard, emerging evidence suggests that inflammation-related markers play a significant role in DFU. One such potential marker, the monocyte-lymphocyte ratio (MLR), has not been extensively studied in relation to DFU. This study aimed to define a connection between MLR and DFU.

**Methods:**

A cross-sectional study was conducted using National Health and Nutrition Examination Survey (NHANES) data from 1999 to 2004. DFU was defined based on survey questionnaires assessing the presence of nonhealing ulcers in the lower extremities for more than 4 weeks in diabetes patients. The MLR was calculated as the ratio of the monocyte count to the lymphocyte count, which was directly obtained from laboratory data files. Logistic regression analysis was performed to assess the relationship between the MLR and DFU. Stratified analysis according to age, sex, body mass index, blood glucose, hemoglobin, and glycated hemoglobin categories was conducted, and multiple imputations were applied to missing data.

**Results:**

In total, 1246 participants were included; the prevalence of DFU was 9.4% (117/1246). A multivariable regression model revealed a significant association between DFU and a 0.1 unit increase in MLR after adjusting for all covariates (adjusted odds ratio=1.16, 95% confidence interval: 1.02-1.33). Subgroup analyses revealed consistent findings regarding the impact of MLR on the presence of DFU (*p* > 0.05).

**Conclusion:**

MLR is significantly associated with DFU in diabetes patients, and can be used as one of the indicators for predicting the occurrence of DFU. MLR assessment may be a valuable component in the follow-up of patients with diabetes.

## 1 Introduction

Diabetes mellitus is a health issue affecting 529 million patients worldwide (1). Alarmingly, up to one-third of these individuals are at risk of developing the diabetic foot ulcer (DFU) at some point in their lives. Furthermore, research indicates that over 15% of DFU eventually lead to lower extremity amputation (2). Various factors contribute to the increased likelihood of DFU development among individuals with diabetes, including prolonged diabetes duration, poor glycemic control, Charcot deformity, diabetic peripheral neuropathy, peripheral arterial disease (PAD), and a history of ulcers or amputation (3–6). The quality of life for DFU patients can be significantly improved through early identification, rapid treatment, and continued foot care.

The presence of inflammation significantly influences the development and progression of DFU (7–9). Clinicians commonly utilize laboratory markers such as white blood cell (WBC) count, C-reactive protein (CRP) level, and erythrocyte sedimentation rate to monitor levels of inflammation (10, 11). There has been a steady increase in research showing that certain inflammatory biomarkers, including CRP, procalcitonin (PTC), malondialdehyde and tumor necrosis factor-alpha, are associated with an elevated risk of DFU over time (9, 12–15). However, despite the close association of inflammatory markers with the diagnosis and prognosis of DFU, there currently lacks a specific inflammatory indicator.

The monocyte-to-lymphocyte ratio (MLR) has emerged as a novel and promising inflammatory marker, obtained by dividing the absolute monocyte and lymphocyte counts in blood samples (16). It has been widely studied in various inflammation-related disorders, such as cancer, tuberculosis and cardiovascular diseases, and has proven to be a reliable biomarker of systemic inflammation (17–19). Elevated MLR has been associated with poor prognosis and accelerated disease progression in several disorders, including acute kidney injury and hematoma after cerebral contusion (20, 21). Recently, some studies have shown that the MLR is associated with the occurrence and progression of diabetes complications, including diabetic nephropathy, diabetic retinopathy (DR), and PAD (7, 22–24).It is reasonable to assume that MLR may play a significant role in the onset and progression of DFU, given the mounting body of research emphasizing its importance in diabetes complications (7, 22–24). However, it is currently unclear whether there is a relationship between MLR and the occurrence and progression of DFU.

In order to comprehensively elucidate this interaction, more research is required to determine the precise connection between MLR and DFU. This study aimed to evaluate MLR’s clinical and predictive value in diabetic patients with DFU. We aimed to clarify the possible function of MLR as a prognostic marker for DFU by assessing MLR levels in a sample of patients and examining their clinical outcomes. This knowledge may have significant effects on early identification, care, and general management of DFU in patients with diabetes.

## 2 Methods

### 2.1 Study design and participants

Data on health and nutrition were gathered from Americans as part of the National Health and Nutrition Examination Survey (NHANES). The participants completed questionnaires on their histories and habits, in addition to physical and laboratory examinations.

We used open data from three NHANES cycles (1999–2000, 2001–2002, and 2003–2004) for the analysis. The NHANES website (www.cdc.gov/nchs/nhanes/) provides further details on the data. To participate in the survey, the subjects were required to undergo a blood test. In-person interviews conducted in the participants’ homes were also used to gather data on basic demographics and medical histories.

A stratified multistage probability survey was used in NHANES research to assess the health and nutritional status of non-institutionalized Americans (25). A mobile examination center conducts home visits, screenings, and laboratory tests as part of the NHANES to gather detailed demographic and health data. The National Center for Health Statistics Ethics Review Committee approved the NHANES, and all participants signed written informed consent forms before participating. No additional Institutional Review Board permission was required for the secondary analysis (http://grants.nih.gov/grants/policy/hs/hs_policies.htm). The NHANES website (http://www.cdc.gov/nchs/nhanes.htm) provides access to the NHANES data.

### 2.2 Study variables and outcomes

MLR stands for monocyte count/lymphocyte count, and laboratory data files in the NHANES can be used to acquire both values directly. Participants were classified as having diabetes if they had any one of the following symptoms: (1) HbA1c ≥6.5%, (2) random blood glucose ≥11.1 mmol/L, (3) fasting blood glucose ≥7.0 mmol/L, (4) utilizing any anti-glycemic medications, (5) ever having been diagnosed with diabetes by a doctor. DFU was identified using survey questionnaires that evaluated the presence of nonhealing ulcers in the lower limbs of individuals with diabetes for >4 weeks. Baseline characteristics included age, sex (male or female), marital status, race/ethnicity, educational attainment, smoking status, body mass index (BMI), HbA1C, blood glucose, hemoglobin (HGB), high-density lipoprotein cholesterol (HDL), total cholesterol, WBC count, and CRP. **Table 1** shows a classification of the necessary baseline characteristics.

**Table 1:** Classification of the necessary baseline characteristics.

Baseline characteristics	Classification
**Marital status**	Married or living with a partner
	Living alone
**Race/ethnicity**	Non-Hispanic white
	Non-Hispanic black
	Mexican American
	Others
**Education level**	Low: Less than 10 years of education (<9 years)
	Medium: 10 to 12 years of education (9-12 years)
	High: More than 12 years of education (> 12 years)
**Smoking status**	Never smoking: less than 100 cigarettes smoked in a lifetime
	Former smokers: >100 cigarettes smoked in a lifetime but no longer smoked
	Current smokers: >100 cigarettes smoked in a lifetime and smoked someday/everyday
**BMI**	Underweight/normal: < 25 kg/m^2^
	Overweight: 25.0–29.9 kg/m^2^
	Obese: ≥ 30.0 kg/m^2^

BMI, body mass index.

### 2.3 Statistical analysis

The statistical software packages R 3.3.2 (http://www.R-project.org, The R Foundation) and Free Statistics software version 1.8 were used for all analyses. Indicators of demographic and clinical characteristics are expressed as means, standard deviations, and frequencies (percentages). Independent and chi-square tests were used to examine the differences between continuous and categorical data. We employed binary logistic regression on single and multiple variables to examine the connection between MLR and DFU in more detail and presented four models for multivariate logistic regression: (1) model 1: unadjusted, (2) model 2: adjusted for sociodemographic variables (age, sex, marital status, race/ethnicity, education level), model 3: adjusted for sociodemographic variables and other variables (*P <*0.05), including HGB and glucose, model 4: adjusted for sociodemographic variables, HGB, glucose, smoking status, BMI, HbA1c, HDL, WBC, and CRP. The association between MLR and DFU was examined using subgroup analysis in relation to age, sex, HGB category (bisection), HbA1c category (< 6.5%, ≥ 6.5%), and glucose levels (< 7 mmol/L, ≥ 7 mmol/L) using a multivariate logistic regression model. A logistic regression model interaction test was performed to examine the odds ratios (OR) between the studied subgroups. On an average, less than 10% of the data were missing. We produced five datasets and combined their analyses to impute missing covariate data using multiple imputations.

## 3 Results

### 3.1 Study population and baseline characteristics

**Figure 1:**
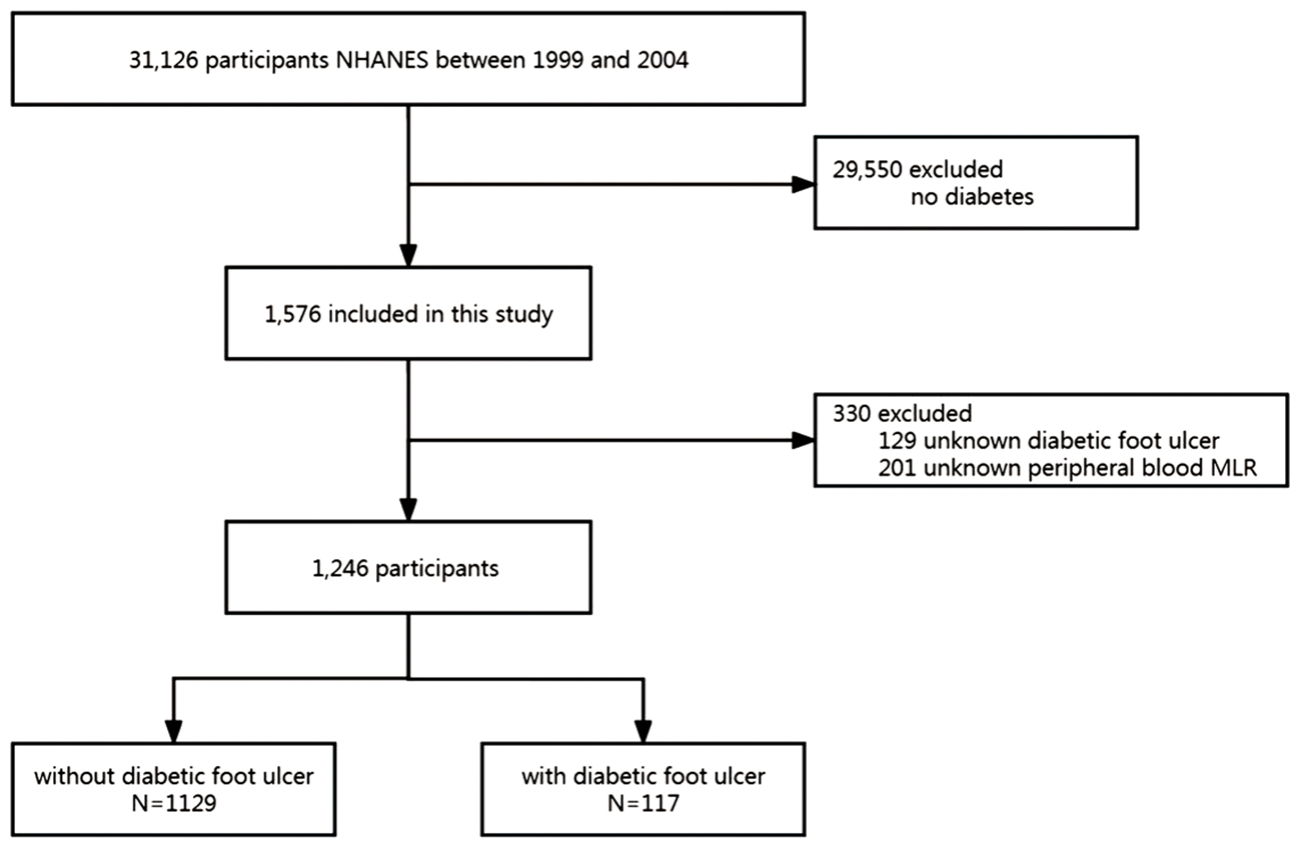
Study flow diagram. NHANES, National Health and Nutrition Examination Survey; MLR, monocyte-lymphocyte ratio.

This study collected data from three NHANES cycles, specifically from the years 1999-2000, 2001-2002, and 2003-2004. The initial pool of potential participants consisted of 31,126 individuals. From this pool, 29,550 participants without diabetes were excluded. Moreover, 129 participants with missing DFU data were excluded. After excluding 201 participants with missing peripheral blood MLR, 1,246 participants remained and were included in the analysis. **Figure 1** presents the inclusion and exclusion criteria and a flowchart outlining the selection process of participants for the study. **Table 2** summarizes the baseline characteristics of both patients with DFU and the non-DFU population (NDFU) in terms of demographics, socioeconomic status, comorbidities, and baseline characteristics. Of the 1,246 participants, 117 (9.4%) were identified as having DFU. Statistical analysis revealed a significant difference in BMI and HGB (hemoglobin) levels between the DFU and NDFU groups, with *p*-values less than 0.05 for both comparisons. Specifically, the DFU group had significantly lower HGB levels (*P* = 0.025) and a larger obese population (*P* = 0.029) than that associated with the NDFU group.

**Table 2:** Baseline characteristics of the study participants.

Variable	OR (%95CI)	*P*
**Age (years)**	1 (0.98-1.02)	0.903
Sex, n (%)		
Male	1	
Female	1.05 (0.95-1.15)	0.355
Marital status, n (%)		
Married or living with a partner	1	
Living alone	1.32 (0.9-1.94)	0.159
Race/ethnicity, n (%)		
Non-Hispanic white	1	
Non-Hispanic black	0.75 (0.43-1.31)	0.312
Mexican American	1.03 (0.66-1.61)	0.896
Others	0.78 (0.35-1.72)	0.534
Education level (years), n (%)		
<9	1	
9-12	1.33 (0.82-2.15)	0.241
>12	1.37 (0.83-2.26)	0.222
Smoking status, n (%)		
Never	1	
Current	1.19 (0.7-2)	0.52
Former	0.88 (0.57-1.35)	0.544
BMI, n (%)		
Underweight/normal	1	
Overweight	0.56 (0.3-1.06)	0.074
Obese	1.08 (0.63-1.87)	0.771
**HbA1c (%)**	1.06 (0.96-1.17)	0.254
**Glucose (mmol/L)**	1.05 (1.01-1.09)	0.02
**HGB (g/dL)**	0.87 (0.78-0.98)	0.025
**HDL (mmol/L)**	0.84 (0.48-1.45)	0.522
**Total cholesterol (mmol/L)**	0.9 (0.76-1.08)	0.269
**WBC, (×10^9^/L)**	1.07 (0.99-1.16)	0.095
**CRP**	1.05 (0.95-1.15)	0.355

BMI, body mass index; HbA1c, glycohemoglobin; HGB, hemoglobin; HDL, high-density lipoprotein; WBC, white blood cell count; CRP, C-reactive protein.

### 3.2 Univariate logistic regression between variables and the presence of DFU

To determine the variables associated with DFU throughout the entire research population, a univariate logistic regression analysis was conducted. A significant positive correlation between HGB, glucose, and DFU was found in the univariate regression analysis (all *P* < 0.05, **Table 3**).

**Table 3:** Univariate analysis for the presence of DFU.

Variable	OR (%95CI)	*P*
**Age (years)**	1 (0.98-1.02)	0.903
Sex, n (%)		
Male	1	
Female	1.05 (0.95-1.15)	0.355
Marital status, n (%)		
Married or living with a partner	1	
Living alone	1.32 (0.9-1.94)	0.159
Race/ethnicity, n (%)		
Non-Hispanic white	1	
Non-Hispanic black	0.75 (0.43-1.31)	0.312
Mexican American	1.03 (0.66-1.61)	0.896
Others	0.78 (0.35-1.72)	0.534
Education level (years), n (%)		
<9	1	
9-12	1.33 (0.82-2.15)	0.241
>12	1.37 (0.83-2.26)	0.222
Smoking status, n (%)		
Never	1	
Current	1.19 (0.7-2)	0.52
Former	0.88 (0.57-1.35)	0.544
BMI, n (%)		
Underweight/normal	1	
Overweight	0.56 (0.3-1.06)	0.074
Obese	1.08 (0.63-1.87)	0.771
**HbA1c (%)**	1.06 (0.96-1.17)	0.254
**Glucose (mmol/L)**	1.05 (1.01-1.09)	0.02
**HGB (g/dL)**	0.87 (0.78-0.98)	0.025
**HDL (mmol/L)**	0.84 (0.48-1.45)	0.522
**Total cholesterol (mmol/L)**	0.9 (0.76-1.08)	0.269
**WBC, (×10^9^/L)**	1.07 (0.99-1.16)	0.095
**CRP**	1.05 (0.95-1.15)	0.355

BMI, body mass index; HbA1C, glycohemoglobin; HGB, hemoglobin; HDL, high-density lipoprotein; WBC, white blood cell count; CRP, C-reactive protein.

### 3.3 Association between MLR and the presence of DFU

The ORs and 95% confidence intervals (CI) for the presence of DFU as assessed by MLR are shown in **Table 4**. In the unadjusted model (model 1), increased MLR was significantly associated with the occurrence of DFU, every 0.1 unit increase in MLR was associated with a 20% increase in the presence of DFU (OR=1.2, 95% CI: 1.08-1.33). In model 2, after adjusting for age, sex, race/ethnicity, education level and marital status, the OR was 1.19 (95% CI: 1.06-1.32); in model 3, after adjusting for age, sex, race/ethnicity, education level, marital status, glucose, and HGB, the OR was 1.15 (95% CI: 1.03-1.29); in model 4, after adjusting for age, sex, race/ethnicity, education level, marital status, glucose, HGB, smoking status, BMI, HbA1C, HDL, WBC and CRP, the OR was 1.16 (95% CI: 1.02-1.33).

**Table 4:** Association between MLR and the presence of DFU.

	Model 1	*P*	Model 2	*P*	Model 3	*P*	Model 4	*P*
	OR (95%CI)		OR (95%CI)		OR (95%CI)		OR (95%CI)	
MLR*10	1.2 (1.08-1.33)	< 0.01	1.19 (1.06-1.32)	< 0.01	1.15 (1.03-1.29)	0.014	1.16 (1.02-1.33)	0.023

Adjusted covariates: Model 1: unadjusted. Model 2: adjusted for sociodemographic variables (age, sex, marital status, race/ethnicity, and educational level). Model 3: adjusted for model 2+ HGB and glucose; Model 4: adjusted for model 3+ smoking status, body mass index, HbA1C, HDL, WBC, and C-reactive protein.

### 3.4 Subgroups analysis of variables that affect the association between MLR and the presence of DFU

**Figure 2:**
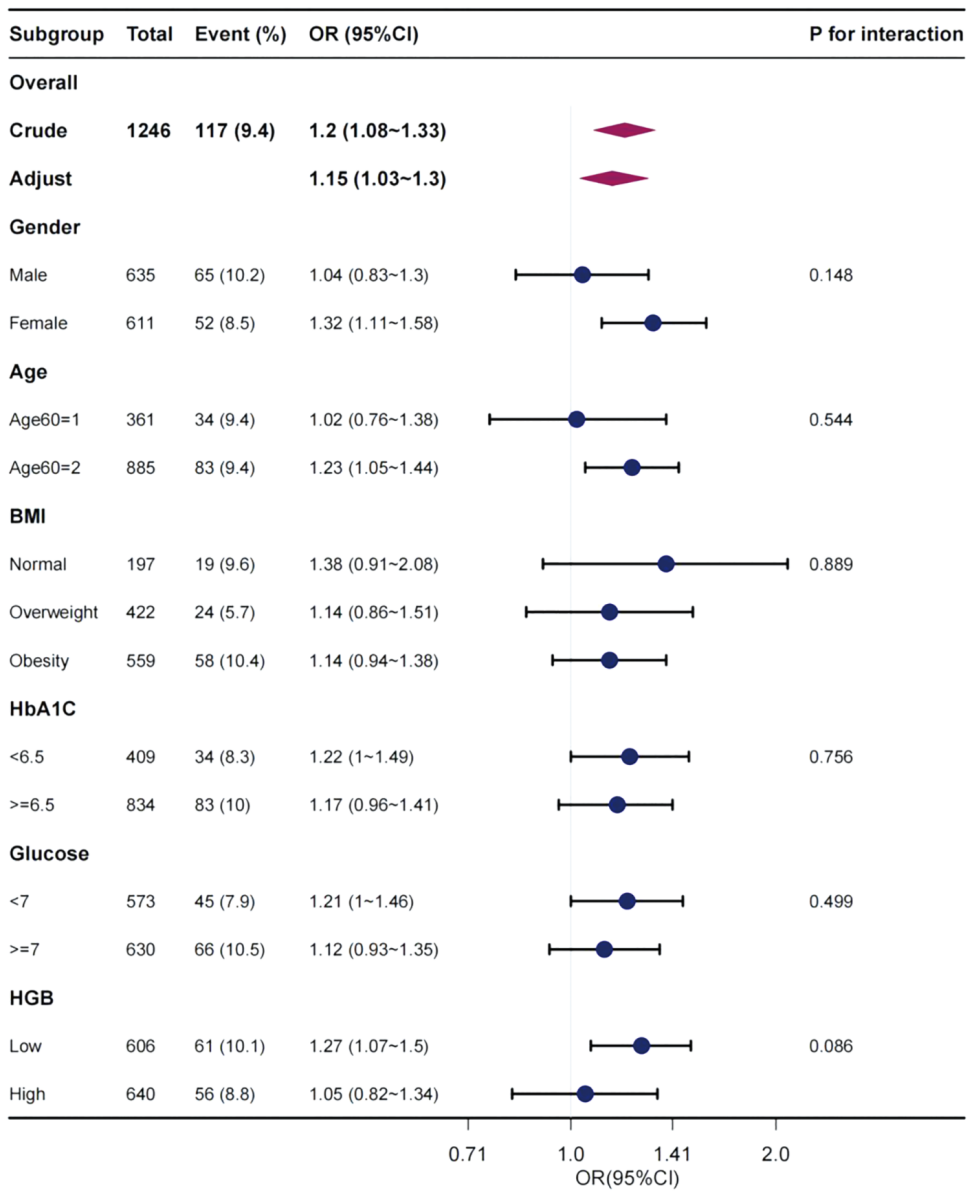
Effect size of MLR on the presence of DFU in subgroups classified in terms of sex, age, BMI, HbA1c, glucose, and HGB. OR, odds ratio; CI, confidence interval, MLR, monocyte-lymphocyte ratio; DFU, diabetic foot ulcer; BMI, body mass index; HbA1C, glycohemoglobin; HGB, hemoglobin.

**Figure 2** explores the relationship between MLR and the existence of DFU in the subgroup analysis stratified by age, sex, BMI category, HbA1C category (< 6.5%, ≥ 6.5%), glucose category (< 7 mmol/L, ≥ 7 mmol/L), and HGB category (bisection). Steady effect size of MLR on the occurrence of DFU in the subgroups. In relation to the presence of DFU, no significant interaction was observed between MLR and sex (*P* = 0.148), age (*P* = 0.544), BMI (*P* = 0.889), HbA1c (*P* = 0.756), glucose (*P* = 0.499), or HGB (*P* = 0.086).

## 4 Discussion

The NHANES database’s data were used to perform the current investigation. An in-depth examination of this database enabled us to examine the connection between the prevalence of DFU and MLR. To our knowledge, our study is the first to show that the incidence of DFU increases with MLR. Specifically, every 0.1 unit increase in MLR was associated with a 20% increase in the presence of DFU. Moreover, our findings revealed a notable link between MLR and DFU. Thus, the MLR may be a useful biomarker for predicting DFU. We considered additional possible variables that could cloud the association between MLR and DFU and discovered that even after controlling for these variables, MLR continued to be strongly correlated with the occurrence of DFU. These findings demonstrate the potential diagnostic value of MLR for identifying patients who are more likely to develop DFU. Early detection and treatment of patients with a high MLR may help delay or even stop the development of DFU, improve patient outcomes, and lessen the burden of crippling diabetic complications.

The onset of diabetes and its associated problems are mostly influenced by pathophysiological circumstances, including inflammation, endothelial dysfunction, ferroptosis and procoagulant imbalance (26–28). These pathophysiological processes interact with metabolic and inflammatory problems in patients with diabetes, causing tissue damage and ultimately leading to consequences, such as PAD, retinopathy, neuropathy, and nephropathy (28).According to several studies, changes in inflammatory biomarkers has been confirmed to be closely related to the occurrence and progression of diabetic complications (7, 22–24). These biomarkers may provide new insights for the early detection and treatment of diabetes and its consequences (27–30).

Systemic inflammation affects neutrophils, lymphocytes, monocytes, and platelets by the fat metabolism, oxidative stress, damaging islet cells and depletion of nutrient (30). During systemic inflammation, neutrophil and monocyte counts are typically high, whereas lymphocyte count may decrease. The neutrophil-to-lymphocyte ratio, platelet-to-lymphocyte ratio, and MLR are indicators of these immune cells. Numerous studies have demonstrated that these markers can predict the presence of systemic inflammation and may be helpful in the diagnosis of various disorders (8, 20, 21, 31). Recently, some studies have shown that the high MLR is associated with the occurrence and progression of diabetes complications, including diabetic nephropathy, DR, and PAD (7, 22–24, 32).

Although inflammation and diabetes or diabetic complications are closely related, limited research has been conducted on the utility of these biomarkers in DFU. Aydın and colleagues confirmed that the systemic immune inflammation index is closely associated with the amputation rate of DFU patients and can be used as a predictive indicator for amputation along with other inflammatory markers (9). Moreover, Umapathy et al. (13) demonstrated that PCT can serve as a valuable indicator for diagnosing type 2 diabetes patients with infected diabetic foot ulcers (with a positive predictive value of 100% and a negative predictive value of 12%). In our study, patients with DFU had a significant high MLR and incidence of DFU increases with MLR. This is similar to the research on MLR in other diabetes complications (7, 22–24). For example, Yue et al. (22) demonstrated that MLR is a risk factor for DR and may be related to the pathophysiology and clinical aspects of DR. Gao et al. (32) explored the association between MLR, the neutrophil-to-lymphocyte ratio (NLR) and the platelet-to-lymphocyte ratio and the risk of non-healing ulceration in patients with type 2 diabetes. They found that after adjusting for confounding variables, MLR and NLR were significantly higher in type 2 diabetes patients with non-healing ulceration. In fact, aberrant glucose metabolism in diabetes leads to altered immunological function of leukocytes (30, 33). This may have an impact on lymphocyte levels, which modulate inflammation. Furthermore, hyperglycemia in individuals with diabetes may increase reactive oxygen species, which can harm lymphocyte DNA by oxidative oxidation and potentially diminish their levels (34). Because of increased lymphocyte death, systemic inflammation frequently leads to reduced lymphocyte levels (35, 36).

This study has several limitations. First, because our study was cross-sectional and relied on data from the NHANES database, we could not prove a causal connection. We were able to establish a connection between MLR and the occurrence of DFU; however, we were unable to establish whether MLR caused DFU or whether DFU caused changes in MLR. Further investigation is required to overcome this limitation, ideally in the form of a prospective study that can offer better proof of causal association. Second, as people may not correctly recall or self-report their diabetes history, self-reported recollections, which was the source of part of the diabetes data, raises the possibility of recall and self-report biases.

This study investigated the relationship between the MLR and DFU incidence. After considering additional variables that may have an impact on the findings, the MLR exhibited a substantial increase in patients with diabetes who had DFU. MLR, obtained from a standard blood test, is a practical and affordable biomarker. Its potential utility during follow-up appointments for diabetes patients is where its significance resides. Further research is required to completely comprehend the underlying mechanism between MLR and DFU.

## Data availability statement

The raw data supporting the conclusions of this article will be made available by the authors, without undue reservation.

## Ethics statement

The studies involving humans were approved by Ethics Committee of Affiliated Hospital of Zunyi Medical University. The studies were conducted in accordance with the local legislation and institutional requirements. Written informed consent for participation was not required from the participants or the participants’ legal guardians/next of kin in accordance with the national legislation and institutional requirements.

## Author contributions

ZL: Writing – review & editing, Writing – original draft, Methodology, Investigation, Formal Analysis, Data curation. YJ: Writing – review & editing, Writing – original draft, Methodology, Investigation, Data curation. ZW: Writing – review & editing, Validation, Supervision, Resources, Project administration, Methodology, Investigation, Funding acquisition.
